# ‘If nurses were in our shoes would they breastfeed their own babies?’ A qualitative inquiry on challenges faced by breastfeeding mothers on the PMTCT programme in a rural community in Zimbabwe

**DOI:** 10.1186/s12884-019-2336-1

**Published:** 2019-05-30

**Authors:** Zibusiso Nyati-Jokomo, Inam Chitsike, Elizabeth Mbizvo, James January

**Affiliations:** 1 Department of Community Medicine, College of Health Sciences, University of Zimbabwe, PO Box A178, Avondale, Harare Zimbabwe; 2 Department of Paediatrics and Child Health, University of Zimbabwe, Harare, Zimbabwe

**Keywords:** HIV/AIDS, Lived experiences, PMTCT, women

## Abstract

**Background:**

The Prevention of Mother to Child Transmission (PMTCT) of HIV programme in Zimbabwe has had remarkable success despite the country’s economic challenges. The aim of this study was to explore the challenges faced by breastfeeding mothers on the PMTCT programme.

**Method:**

Narratives from 15 women (age range 19–35 years) were collected at two rural health facilities in Zimbabwe through in-depth interviews over a period of 6 months. Thematic analysis was used to describe breastfeeding mothers’ experiences and challenges of being on the PMTCT programme.

**Results:**

The findings suggest that breastfeeding women on the PMTCT programme face challenges that include internal, external and institutional stigma and discrimination. Women reported a sense of powerlessness in decision making on following through with the PMTCT programme and were ambivalent regarding disclosure of their HIV status to their partners and significant others.

**Conclusion:**

HIV and AIDS programmes should pay attention to women’s readiness for interventions. There is need to understand women’s life experiences to ensure informed and targeted programming for PMTCT.

**Electronic supplementary material:**

The online version of this article (10.1186/s12884-019-2336-1) contains supplementary material, which is available to authorized users.

## Background

Although prevention of mother-to-child transmission (PMTCT) services have over the years been expanding dramatically with data between 2002 and 2013 showing that new infections among children dropped by more than 50%, there remains a treatment gap due to several reasons [1]. The PMTCT cascade entails a chain of intricate steps such as counselling and testing, disclosure, and treatment. Furthermore, distance to health facility, stigma, food insecurity and negative attitudes of health services providers towards women act as barriers to recommended guidelines for women on PMTCT [2, 3].

The World Health Organization (WHO) guidelines recommend lifelong ART (Option B+) to all HIV positive women who are pregnant or breastfeeding regardless of CD4 count or clinical staging to reduce vertical transmission. In 2013, Zimbabwe adopted Option B+ as a means of ensuring elimination of pediatric HIV infections. The percentages of pregnant mothers in the country who received ART to reduce the risk of mother to child transmission of HIV (MTCT) reached 93% in 2013 and 86% of babies born to HIV positive mothers were initiated on ART [4]. Thus, with the newly recommended Option B+ regimen, Zimbabwe has seen an expansion in access to ART for pregnant women and breastfeeding mothers [4]. With the demonstrated potential of Option B+ there are new challenges such as acceptability of ART, self and community stigma, inadequate counselling and non-adherence [5, 6]. However, there is paucity of data on the experiences of mothers on PMTCT especially during the postnatal period in Zimbabwe. Although, the adoption by Zimbabwe of Option B+ is likely to see major strides towards the elimination of paediatric HIV infections, adherence to this new drug regimen and adherence to infant feeding guidelines by mothers during the postnatal period remains unclear. It is therefore critical to understand lived experiences of women on PMTCT to make informed policy decisions.

PMTCT programmes face a plethora of challenges which include access to health services, long waiting hours, community stigma, disclosure, and adherence which underscores the need to get a deeper understanding of how the women are receiving and experiencing the programme [7–12]. This study therefore aimed to explore HIV infected breastfeeding women’s experiences of the PMTCT programme in rural Zimbabwe.

## Methods

### Research design

This paper reports results from part of a larger study on socio-cultural realities of following through with PMTCT in the Chiota rural community in Zimbabwe [13, 14]. Results from this study were reported using the Consolidated Criteria for Reporting Qualitative Research (COREQ) [15]. A descriptive phenomenological study design was used to understand the experiences of women living with HIV on the PMTCT programme. This design aims to offer insights into how people in given contexts make sense of a certain phenomenon which relate to experiences of personal significance. Qualitative methods were preferred for the study for their strength in providing rich data on lived experiences and taking cognizance of participants’ voices. The researchers engaged with breastfeeding women as they described their experiences of being on the PMTCT programme. The women were interviewed once by two interviewers in each session whilst the third interviewer took notes.

### Participants and setting

The registers at two rural health facilities were used as the sampling frame targeting all postpartum HIV positive mothers. The two rural health facilities had a combined total of 108 women on PMTCT. The purposive sampling technique was utilized to recruit participants who were reported by the nurses to be having difficulties accepting their HIV status and adhering to Option B+. Purposive sampling has its focus on specific characteristics which in this study were rural health facilities that offered PMTCT and breastfeeding mothers who were facing challenges with regards to adherence to PMTCT. The selection criteria were based on having a baby who was at least 2 months, breastfeeding, able to give consent and not seriously ill. The nurses had initially identified 18 women who were having adherence challenges. Of the 18, 15 consented to the study, whilst two were lost to follow up and one woman declined to participate citing a busy schedule. Participation was voluntary and participants were recruited at two government primary healthcare facilities as they came to collect their monthly supplies of medication or when they came for the child welfare clinics. Stigma surrounding HIV and AIDS is still very strong in the area under study such that efforts to recruit mothers from the community became futile, hence the resolution to recruit from the health facilities [13].

### Authors’ positionality

The position of the researcher within the research process is vital. Authors were aware that as researchers they could shape the research process and their own experiences may have a bearing on the knowledge production and presentation. Data were collected by one male (JJ) and three female researchers (ZNJ, IC, EM) who had doctoral level qualifications in psychology, social science, pediatrics, and public health respectively. The researchers had sufficient understanding of the local context, recognized informal institutions which facilitated easy access into the community. They had also worked extensively in Chiota community and had established rapport and trust with the community members. They could easily interpret culturally coded observation. As women and mothers, the researchers were privy to the challenges that affect mothers during the postnatal period. The missing link could have been the experiences of living with HIV. The other male team member who was a psychologist, provided guidance in terms of conduct of the interviews, understanding the women’s behavior, emotions and offering counselling where it was needed. Using multidisciplinary researchers was also meant to enhance the reliability and validity of the findings. All the team members were conversant with the Shona traditions and the multidisciplinary team strengthened the data analysis. It should be acknowledged that subjectivity is always present in qualitative research. To minimize subjectivity, the researchers went into the field with as few preconceptions as possible. This enabled the researchers to conduct a reliable qualitative inquiry which was devoid of past experiences and prejudices.

### Data collection

The purpose of the study was explained to the participants prior to data collection and they were asked to describe their experiences and subjective feelings following initiation on the Option B+ regimen. Data were collected through unstructured in-depth interviews where mothers narrated their experiences of being on the PMTCT programme. This method enabled the collection of personally salient data with some richness and depth. Interviews were conducted in the local Shona language and transcribed verbatim. Only the participants and the researchers were present during the interviews. Each interview lasted for an average of 45 min to 1 h. Data were collected over a period of 6 months.

### Ethical considerations

The study protocol was reviewed and approved by both the Joint Parirenyatwa Hospital and College of Health Sciences Ethics Committee (JREC Ref 4/14) and the Medical Research Council of Zimbabwe (MRCZ/A/1819). Permission to carry out the study was obtained from the Ministry of Health and Child Care, Secretary for Health, Provincial and District Medical Health Directors of Mashonaland East and the Nurses in-charge of the 2 Chiota Rural Health Clinics who assisted in the identification of the mothers. The purpose of the study was explained to the participants. Written and verbal informed consent was given by all the participating mothers. The interviews were conducted in a private room. To avoid coercion, the health care providers did not directly participate in the study.

### Data analysis

The conversations were tape recorded after obtaining the participants’ permission. The interview guide was created in English by the principal investigator (ZNJ) and the other researchers (JJ, IC and EM) revised it to ensure that it answered the research objectives. Since the interview was conducted in Shona, it was sent to the Department of Linguistics at the University of Zimbabwe for translation by a senior lecturer. The researchers who were the originators of the research had to back translate it into English to ensure that it retained its meaning (see Additional files 1 & 2). The tool was pretested at a health facility which was similar to the study areas. The pretest showed that the tool solicited the information that addressed the objectives of the study. One probe on cultural practices was included after the pretest. After data collection, the transcripts from the audio recordings were typed in Shona and then translated into English before coding and analysis was done. The authors are all fluent in Shona and they individually translated the transcripts and they exchanged their translations and reached consensus. The translations did not have any variations and this ensured that there was no difference in interpretation of the data. The authors also translated the excerpts in the results section. Pseudonyms were used when quoting information during data analysis to maintain anonymity.

Data were analyzed thematically [16]. The first step was to summarize each participant’s story followed by analyzing their biographic data. This was followed by coding of significant statements. Three data coders coded the data. Emerging codes were catalogued and patterns in the codes were noted. This was followed by grouping the codes into themes. Quotable quotes were noted whilst continuously interrogating the data. Some emergent themes were further grouped into superordinate and subordinate themes with the final set of themes being summarized and tabulated with evidence from the text supported by quotes. The coded data were independently cross checked by two authors to ensure consistency. The preliminary findings of data were shared with participants.

## Results

### Respondent characteristics

Chiota is a Shona speaking community and all the mothers were of Shona origin. The majority of the participants lived within a 5 km radius to the health facilities with a few living within a 10 km radius. The women’s ages ranged from 19 to 37 years. Two thirds of the women were married, and the other women were either single or widowed. Regarding religion, 13 out of the 15 participants identified themselves as Christians with the remaining 2 stating that they were not affiliated to any religion. Education level was generally high with most of the participants having attained secondary level of education. Thirteen women were unemployed with the remaining 2 employed, both as domestic workers. Table 1 shows the characteristics of the participants.

**Table 1:** Characteristics of participants *N* = 15

Characteristics	Frequency
Age range (years)	
19–24	3
25–30	8
31–37	4
Marital status	
Single	3
Married	10
Widowed	2
Highest level of education	
Primary	3
Secondary	12
Employment status	
Employed	2
Unemployed	13

The analysis resulted in 4 main themes and 12 sub-themes. The superordinate themes were (1) Stigma and discrimination, (2) Fear of HIV transmission to the baby, (3) Family and Power dynamics, and (4) Uncertainty about Antiretroviral (ART) side effects (See Table 2).

**Table 2:** Themes and the sub-themes resulting from the data analysis

Superordinate theme	Subordinate themes
Stigma and Discrimination	Internal/anticipated self-stigma Institutional stigma (poor professional behavior) External/Community stigma
Fear of HIV transmission to baby	Mistrust of mother’s milk, distrust of nurse advise, community advice
Family and Power Dynamics	Disclosure and influence of significant others Fear of divorce
Uncertainty about drug side effects	Fear of prolonged use of drugs Fear of unsustainability of ART

#### Theme 1: stigma

Our findings showed that living with HIV was still stigmatized in the community as all the participants (*n* = 15) reported having experienced some form of stigma. The narratives from the women indicated that the disease was still associated with deviant behaviour and this led to internalized stigma.

##### Sub-theme 1: internal stigma

Most of the women reported that they experienced low self-esteem [anticipated stigma] because of being HIV positive. The theme appeared universal as mentioned by most of the mothers. They felt that their condition was the talk of the community and they were no longer free to interact with other members of the community. One respondent remarked:

‘…*whenever I approach other women they change their discussions and you can tell that they were talking about you. Some even treat you as if you are a charity case as they will offer to carry your luggage or offer to buy you fruits’* (A participant within the age range of 20-25 years , married).

In relation to self-stigma one woman said:

‘…*it is as if I have a stench…I guess I deserve this because I used to despise people who were living with HIV. …I never thought it would happen to me’* (A participant who was single within the age range of 20-25 years).

##### Sub-theme I.2 institutional/health services stigma)

Two thirds of the women felt that health care providers were treating them differently from the rest of the other health care seekers. The women were asked to sit in their own queue and it became obvious that they were HIV positive. Respondents reported that precedence was given to other patients and they experienced long waiting hours at the health facilities.

*‘…you are required to report to the health facility at 8am and yet they start serving us after lunch when they have cleared all other patients. They hide behind the fact that they need to counsel us as a group’* (A participant within the age range of 30–35 years).

##### Sub-theme 1.2.1 poor professional behaviour

Some reported that health care providers exhibited poor professional behaviour as they blatantly discussed the women’s issues in the presence of other health care seekers. Across all interviews the women narrated how the health professionals had failed to observe confidentiality as they would ask them questions related to their HIV statuses when the other patients were listening. One of the HIV breastfeeding women attributed this to shortage of space at the health facilities.

*‘…may be it is also unfair to blame the health professionals as they are incapacitated when it comes to space and privacy’* (A participant who was single within the age range of 20–25 years).

##### Sub-theme 1.2.3 timing for HIV services

HIV services were offered on the last Thursday of every month. Setting aside Opportunistic Infection (OI) days made the whole community aware of the women’s conditions. All HIV positive patients who were on ART were collecting their ART supplies on this day and the health facilities were a hive of activity on these particular days. The community were also taking advantage of these days to come and sell their wares which included fruits, vegetables and second-hand clothing.

One of the women commented *‘…some people are now coming to the clinic on such days to spy and get the juiciest gossip about people living with HIV’.*

##### Sub theme 1.2.4 the packaging of ART tablets

The packaging of the ART tablets which is in big boxes made it obvious that the women were collecting their monthly supplies. All the mothers felt that they were not empowered to make their own decisions pertaining to opting out of the PMTCT programme. HIV testing, ART initiation and the infant feeding plan of exclusive breastfeeding was dictated. The following excerpt illustrates this:

‘*They (healthcare providers) have no confidentiality as they coercively test us, prescribe ways to breastfeed and distribute our drugs in full view of the community members. We are made to queue for a long time and when they want to attend to us they will shout “Vemapiritsi huyayi” (those for tablets come and collect your drugs’* (A participant within the age range of 30-35 years, married).

##### Sub theme 1:3 community (external stigma) and discrimination

All the participants felt that HIV stigma in the community was still rife. Access to free ARVs for all the people living with HIV was reportedly viewed by the community members as discriminatory. The women reported that the community sentiments were that people living with HIV were being prioritized and getting the best services (free drugs) when other people were failing to get drugs for more genuine ailments like non-communicable diseases which include high blood pressure and diabetes.

‘*Since the advent of ART, people in the community are no longer sympathetic as they believe that our disease is self-inflicted…some feel we are benefitting more…even my relatives seem to share these sentiments’* (A participant within the age range of 25-30 years, married)

#### Theme 2: fear of HIV transmission to the baby

All the participants feared that they would at some stage infect their babies. Mothers felt that the counselling they received at the health facilities was not convincing enough that their babies would remain negative especially after the recommended exclusive breastfeeding for the first six months. Two thirds of the mothers had had their infants tested for HIV and they all said their babies had tested negative. This further made them fear that their children would seroconvert if they continued breastfeeding.

##### Sub theme 2:1 fear of infecting the baby through mother’s breast milk

Across all interviews the participants feared that the virus in their milk would infect their babies. Mothers showed universal feelings of despondency as they mistrusted breastfeeding their babies. Two thirds of the mothers confessed attempting to minimize breastfeeding their babies but due to economic hardships they were forced to breastfeed when they were unable to offer alternative feeds. A distraught mother asserted;

‘*I do not trust my milk at all and I minimize giving my baby breast milk when I have alternative feeds’* (A married participant within the age range of 25-30 years).

##### Subtheme 2.2. Distrust of nurses advice concerning breastfeeding

The mothers mistrusted the advice they received from the nurses on the safety of breast milk. To further confound this were the voices from the community members and significant others who discouraged the HIV positive mothers from breastfeeding. All the women suggested that the beliefs in the community were that an HIV infected mother’s milk was unsafe for infant feeding. Basing on this strongly held community belief, one respondent said;

‘*The nurses are cruel. They ask us to breast feed our babies when they are fully aware of our statuses. If they were in our shoes would they breastfeed their own babies?’* (A widowed participant within the age range of 35-40 years).

#### Theme 3: family and power dynamics

Across all interviews, the participants reported that being diagnosed HIV positive had negatively impacted on their marital relationships. Most of the women felt that they had behaved irresponsibly by having babies when they knew that they were HIV positive. A few women reported being pressured to have babies to dispel the suspicions of being HIV positive. Six of the fifteen participants had however known of their HIV status during the routine antenatal care and they were still coming to terms with an HIV positive diagnosis. Some of the women had not planned to have the babies blaming the failure of family planning method for their unplanned pregnancies. All the women in our sample blamed their partners for having infected them. The common statement from the women was *‘…men are irresponsible beings who have got us into this mess’.*

##### Sub theme 3:1 disclosure and influence of significant others

Disclosure of HIV status was still problematic as only 7 out of the 15 women had disclosed to their husbands, a few women had disclosed to some trusted family members and whilst the others had kept their serostatus as a secret although they suspected that due to lack of confidentiality at the health facilities the community members and significant others suspected that they were HIV positive. The following are the mothers’ narratives of their experiences:

‘*When I was diagnosed HIV positive during ANC, I (hypothetically) asked my husband for his reaction…he became so livid… I hide my medication in the garden… I wish he could go and get tested and access medication as he is losing weight’*. (A married participant within the age range of 30-35 years).

‘*My mother-in-law never liked me as she often referred to me as a prostitute…and she will send me packing upon realization of my status. She has been giving my child so many traditional herbs and I cannot say anything’.* ( A married participant within the age range of 25-30 years).

##### Sub theme 3:2 fear of divorce and rejection

Most of the married participants feared that their partners would divorce them if they insisted on condom use to protect their babies. The women mistrusted their partners as they alleged that they were likely to be engaging in extramarital affairs which had brought about the infection. Most participants also felt that the significant others like mothers-in-law, aunts and other close relatives could influence their husbands/partners to divorce them. The fear was aggravated by the fact that being HIV positive diminished chances of ever getting a marriage partner in the event of being divorced.

‘*My husband knows that I am HIV positive but he insists on having unprotected sex and often threatens to divorce me if I resist’. (A married* participant within the age range of 30-35 years).

*‘I knew I was HIV positive before I got pregnant but due to the pressure from my in-laws I had this baby… am hoping that she does not get infected during breastfeeding as I would be asked to leave. My husband died when I was 3 months pregnant and I am staying at this homestead because of this baby. If I am asked to leave I would be destitute’*. (A widowed participant within the age of 25-30 years).

#### Theme 4: uncertainty about drug side effects

Mothers who were newly diagnosed during ANC were not very keen to be commenced on lifelong treatment as they felt that they had not been given a chance to opt out. On the other hand, some mothers who already knew their status felt that this was the best package which made easy access to ART. Discussions with all the mothers revealed that there was an inherent fear of side effects from the ARVs.

##### Sub theme 4: 1 fear of prolonged drug use

Prevention of Mother to Child Transmission entails being initiated on Option B+. This is mandatory for all HIV positive breastfeeding mothers. During the discussions, the breastfeeding mothers kept referring to the side effects from the drug Stavudine which had disfigured HIV patients through lipodystrophy (build up or loss of body and uneven distribution of fat around the abdomen, neck, legs and buttocks) [17]. They were not confident that the new drug regimen was devoid of the side effects.

‘*When I came to register my pregnancy, I was tested and found positive and immediately commenced on ART when I was not even sick. This is a lifelong treatment and I am not sure if I am prepared to continue after breastfeeding as I have seen some people developing hunch backs because of ARVs’* (A married participant within the age of 20-25 years).

##### Sub theme 4: 2 uncertainty about sustainability

Most mothers feared that ART provision would not be sustainable under the prevailing economic crisis in the country and they would be worse off if they had to stop accessing the drugs.

*‘What if these free drugs run out or we are asked to pay for them which we cannot obviously afford’* (A participant within the age range of 25-39 years, married).

## Discussion

In this study the main question under discussion centered around understanding the lived experiences of breastfeeding women on the Option B+ programme in an economically disadvantaged rural setting. Our findings revealed that women had concerns with regard to stigma and discrimination, fears surrounding infecting their babies with HIV, strained marital relationships, and were uncertain about ART side effects.

In relation to stigma, the sentiments expressed by women in our study are a clear indication of internalized stigma, institutional and community stigma. This is similar to findings by Schechter et al. (2014) in Cote d’Ivoire and Uganda where it was shown that women experienced depression when they became aware of their HIV positive status [6, 18]. A study in Mozambique reported unintentional biases among healthcare providers which were a frontier of stigmatizing HIV positive women a similar finding to our study which highlighted institutional stigma as a serious challenge for mothers on the PMTCT programme [19]. A study conducted in Ethiopia further confirmed that stigma and discrimination by the health care providers was associated with poor adherence [20]. Women in our study reported a sense of powerlessness which was compounded by unequal relations with the health care providers, a similar finding to a study in Tanzania by An et al. (2015) which found that women were not at liberty to opt out for compulsory testing [21]. The fear of negative consequences and the stigmatization of HIV by the community were found to be a barrier to disclosure in our study which was also found in a systematic review on ART use among postpartum women [22]. In Botswana, women also kept their HIV status as a well-guarded secret in the communities where they lived as they feared being stigmatized and discriminated against [22, 23]. Community involvement in HIV and AIDS prevention efforts in our study was said to be lacking contrary to Campbell and colleagues in 2013 in Zimbabwe who postulated community involvement in HIV prevention and mitigation as a critical enabler of an HIV and AIDS response [25]. Community readiness which include identification of existing obstacles to programme success should be prioritized in all HIV and AIDS programme interventions.

Other studies have also shown that women on the PMTCT programme experience fear especially relating to breastfeeding their babies and that healthcare providers oftentimes fail to adequately consider factors such as the women’s lived experiences, their preferences, social networks and lay knowledge, all of which have potential for inhibiting effective participation in PMTCT programmes [25–27]. In a study that was conducted in Zimbabwe, HIV positive women were reported to suffer anticipatory grief especially when their child was also HIV positive [28]. Additionally, the choice of feeding strategies like exclusive breastfeeding was found to be a cause of distress among women as it was susceptible to judgment and association with HIV and AIDS [23, 24, 29]. The lack of economic empowerment also left women with no choice of giving their babies alternative feeds as they were not employed and they depended on their partners and extended family for sustenance.

Our findings also suggested that women felt that being HIV positive strained relationships within their marriages. Women faced a dilemma in disclosing to their partners and feared divorce and violence following disclosure. Disclosure of one’s HIV status can improve uptake and retention in PMTCT services, yet it has been found to be a serious challenge for most women. A systematic review which evaluated disclosure rates revealed that HIV serostatus disclosure ranged from 5.0 to 96.7% and women were more likely to disclose to their partners than to family members [30]. Another study on non-disclosure of HIV status among Sub Saharan migrant women also identified sigma as the chief reason for non-disclosure [31]. A study in Botswana revealed that women faced a dilemma of disclosing their HIV status especially to their partners and preferred to disclose to their own mothers and sisters [23]. In Mombasa women were reported to have faced intimate partner violence and divorce after HIV disclosure [29]. These findings relate to our study which found disclosure among HIV positive women to be one of the greatest challenges. The commonly cited barriers for non-disclosure of HIV status by women on the PMTCT programme included fear of abandonment by significant others and divorce by husbands a finding confirmed by Gari and Habte, (2010) in Ethiopia [32]. Shamu and colleagues reported intimate partner violence following disclosure of HIV status among pregnant women in Zimbabwe [33]. The narratives by Maman, Rooyen, and Groves, in South Africa on disclosure reported negative reactions from a partner upon disclosure which is congruent with our findings [34].

Womens’ fear of the long-term effects of ART use found in a study was similar to that found in Nigeria [35]. The introduction of the WHO recently recommended Option B+, for all pregnant and breastfeeding mothers irrespective of CD4 count and clinical staging was a cause of concern for mothers who were not sure of its sustainability and side effects.

### Study limitations

The study was conducted at health facilities which could be intimidating for mothers to express especially the institutional challenges they were encountering. The study also focused only on women with challenges, calling for further studies to explore experiences of other women to draw comparisons. The study findings cannot be generalized due to the small sample size.

## Conclusion

This study showed that breastfeeding mothers on the PMTCT programme face numerous challenges which include fear of disclosure, stigma, discrimination and poor patient-provider relationships. Availing the Option B+ regimen is not enough if contextual factors along the continuum of care are not considered. Taking into cognizance women’s experiences might lead to better programming and success of the PMTCT programme as Zimbabwe strives towards eliminating HIV in infants and keeping mothers alive.

## Additional files

Additional file 1: Interview guide for HIV positive breastfeeding women in English. (DOCX 15 kb)

Additional file 2: Interview guide for HIV positive breastfeeding women in Shona. (DOCX 15 kb)

## Abbreviations

AIDS: Acquired Immunodeficiency Syndrome
ANC: Antenatal Care
ART: Antiretroviral Therapy
HIV: Human Immunodeficiency Virus
JREC: Joint Parirenyatwa Hospital and College of Health Sciences Research Ethics Committee
MRCZ: Medical Research Council of Zimbabwe
MTCT: Mother to Child Transmission
OI: Opportunistic Infections
PMTCT: Prevention of Mother to Child Transmission
