# Supplementary material for: ‘If nurses were in our shoes would they breastfeed their own babies?’ A qualitative inquiry on challenges faced by breastfeeding mothers on the PMTCT programme in a rural community in Zimbabwe
Source: BMC Pregnancy Childbirth. 2019 May 30;19:191. doi: 10.1186/s12884-019-2336-1 (PMC6543664; doi:10.1186/s12884-019-2336-1)
Supplement: Supplementary file 1 — Interview guide for HIV positive breastfeeding women in English. (DOCX 15 kb) [file 12884_2019_2336_MOESM1_ESM.docx]

**Additional File 1**

**Title: Interview guide for HIV positive breastfeeding women in English
 Brief description of the interview**

HIV positive women attending 2 rural health facilities who were identified by the nurses as being similar in that they were having difficulties in accepting their HIV status and adhering to Option B+ were recruited. The selection criteria was based on having a baby who was at least 2 months, breastfeeding, able to give consent and not seriously ill. The nurses had initially identified 18 women who were having adherence challenges. Of the 18, 15 consented to the study, whilst two were lost to follow up and one woman declined to participate citing a busy schedule.

The trained interviewers introduced themselves and effectively informed the interviewees about the purpose of the study, that it was not for personal gains and that the information that they would provide would only be accessed and used by the researchers and that it would not be linked to them in anyway. Participation was voluntary and the women individually provided written informed consent. The interview guide questions below ensured that the same general areas of information were collected from each interviewee and probes were used to elicit further information.

**General questions used to elicit information were:**

1. Can you share your experiences of living with HIV. (Do not give your real name, but tell us about your real age)
2. What information are you given on infant feeding practices by *;*
3. The health facility staff
4. The community
5. Relatives and significant others
6. What is your understanding of exclusive breastfeeding?
7. What is your opinion on exclusive breastfeeding by mothers living with HIV?
8. What are the barriers to exclusive breastfeeding by HIV positive mothers?
9. What other challenges do you face?

**Probes used for eliciting more information related to:**

- Resources- ability to give alternative feeds to the baby
- Informed choices on feeding practices
- Adherence to exclusive breastfeeding
- Cultural practices regarding infant feeding
- HIV and Stigmatisation
