# Supplementary material for: ‘If nurses were in our shoes would they breastfeed their own babies?’ A qualitative inquiry on challenges faced by breastfeeding mothers on the PMTCT programme in a rural community in Zimbabwe
Source: BMC Pregnancy Childbirth. 2019 May 30;19:191. doi: 10.1186/s12884-019-2336-1 (PMC6543664; doi:10.1186/s12884-019-2336-1)
Supplement: Supplementary file 2 — Interview guide for HIV positive breastfeeding women in Shona. (DOCX 15 kb) [file 12884_2019_2336_MOESM2_ESM.docx]

**Additional File 2**

**Title: Interview guide for HIV positive breastfeeding women in Shona
 Brief description of the interview**

**Mibvunzo yanamai vanorarama neutachiwana vachiyamwisa vana
 Tsanangudzo**

Tsanangudzo iyi yanga yakanangana nanamai vanorarama neutachiwana vanga vachitsvaga rubatsiro kuzvipatara mbiri. Vanga vaonekwa nevanamukoti kuti vanga vachisangana nematambudziko pakurarama neutachiwana. Kusagamuchira kwavo kurarama neutachiwana zvaiitisa kuti vasateedzere chirongwa cheOption B+. Vese vana mai vakasarudza kupinda muchirongwa ichi vasina kumanikidzwa huye vanga vachiyamwisa vana vanemwedzi miviri zvichiyenda mberi vasingarware. Vanamukoti vanga vasarudza vanamai vanosvika 18. Kunevakasarudzva, 15 ndivo vakapinda muchirongwa, mumwe chete akaramba achiti anga asina nguva, vaviri avanakuwanikwa.

Vaongorori vakatsanangura maerano nechirongwa, vachisimbisa huye vachizivisa vanamai ava kuti zvinhu zvavaizotaura zvaizochengetedzwa pakahwanda. Vana mai vakaudzwa zvekare kuti vaibvimidzwa kurambe kuve nhengo yetsanangudzo iyi zvisingazovanetsa. Mibvunzo inoteera ndiyo yakashandisirwa vese vakasarudza kupindamuchirongwa ichi.

***Mibvunzo yakashandiswa kutsvaga ruzivo***

1. Tsanangura maererano nemararamiro ako neutachiwana we HIV (Usataure zita rako chairo , unogona kutiudza makore ako chaiwo)
2. Ndedzipi dzidzo dzamunopuwa maererano nekupa vacheche zvekudya ne ;
3. Vashandi vezveutano
4. Vanhu vanogara munharaunda
5. Veukama neshamwari
6. Munonzvisisei pamusoro wekuyamwisa mwana mucheche mukaka wamai chete usingamupe chero chinhu?
7. Munofungei pamusoro wekuyamwisa mwana mukaka wamai vanorarama neutachiwana?)
8. Ndeapi matambudziko amunosangana nawo pakuyamwisa mwana muchirarama neutachiwana?
9. Chitsanangura ese mamwe matambudziko amunosangana nawo anosanganisira kurarama kwenyu neutachiwana

**Bvunzisisai maerano ne zvinoteera kana zvisina kutaurwa**

- Kuwanikwa kwezvokupa mwana
- Kuita sarudzo nezvamunoda kupa vana vacheche
- Kuteedzera yambiro yamunopiwa maererano nezvekuyamwisa vacheche
- Tsika nemagariro maererano nekupa vacheche zvekudya
- Kurarama neutachiwana nerusarura.
